# Long-term effects of laparoscopic sleeve gastrectomy versus roux-en-Y gastric bypass for the treatment of Chinese type 2 diabetes mellitus patients with body mass index 28-35 kg/m^2^

**DOI:** 10.1186/s12893-015-0074-5

**Published:** 2015-07-22

**Authors:** Jingge Yang, Cunchuan Wang, Guo Cao, Wah Yang, Shuqing Yu, Hening Zhai, Yunlong Pan

**Affiliations:** Department of General Surgery, First Affiliated Hospital of Jinan University, Guangzhou, 510630 China

**Keywords:** Bariatric surgery, Roux-en-Y gastric bypass, Sleeve gastrectomy, Type 2 diabetes, Mild obesity

## Abstract

**Background:**

To compare long term effects of two bariatric procedures for Chinese type 2 diabetes mellitus (T2DM) patients with a body mass index (BMI) of 28-35 kg/m^2^.

**Methods:**

Sixty four T2DM patients with Glycated hemoglobin A1c (HbA1c) ≧ 7.0 % were randomly assigned to receive laparoscopic sleeve gastrectomy (SG) or Roux-en-Y gastric bypass (RYGB) procedure. Weight, percentage of excess weight loss (%EWL), BMI, waist circumference, HbA1c, fasting blood glucose (FBG), and C-peptide were measured. Serum lipid levels were also measured during three-year postoperative follow-up visits.

**Results:**

Fifty five patients completed the 36-month follow-up. Both groups had similar baseline anthropometric and biochemical measures. At the end point, 22 patients (78.6 %) in SG group and 23 patients (85.2 %) in RYGB group achieved complete remission of diabetes mellitus with HbA1c < 6.0 % (*P* = 0.525) and without taking diabetic medications, and 25 patients in each group (89.3 % vs. 92.6 %) gained successful treatment of diabetes with HbA1c≦6.5 % (*P* = 0.100). Change in HbA1c, FBG and C peptide were comparable in the two groups. The RYGB group had significantly greater weight loss than the SG group [percentage of total weight loss (%TWL) of 31.0 % vs. 27.1 % (*P* = 0.049), %EWL of 92.3 % vs. 81.9 % (*P* = 0.003), and change in BMI of 11.0 vs. 9.1 kg/m^2^(*P* = 0.017), respectively]. Serum lipids in each group were also greatly improved.

**Conclusion:**

In this three-year study, SG had similar positive effects on diabetes and dyslipidemia compared to RYGB in Chinese T2DM patients with BMI of 28-35 kg/m^2^. Longer term follow-ups and larger sample studies are needed to confirm these outcomes, however.

## Background

Obesity and type 2 diabetes mellitus (T2DM) are two of the most common metabolic disorders in the world. Both have significantly increased during the last decades [1, 2]. In China, the prevalence of obesity and T2DM is similar to the worldwide statistics. In China it is estimated that the number of people with diabetes was 98.4 million 2013 and will reach 142.7 million by 2035 [2].

Bariatric procedures are superior to conservative therapies in managing T2DM [3, 4]. Roux-en-Y gastric bypass (RYGB) is the most commonly supported procedure that can cure most T2DM in morbidly obese patients [3, 5, 6]. Sleeve gastrectomy (SG), a novel technique, is highly effective in the treatment of severe or morbid obesity [7, 8]. It is still controversial, however, whether SG has the same positive outcomes on T2DM in mild obese patients compared to RYGB [9, 10]. Importantly, most of the Chinese T2DM patients that have been studied have BMI less than 35 kg/m^2^ and are newly detected diabetes cases with short disease durations [11]. Other relevant reports about long term effects of SG on Chinese diabetes with BMI of 28-35 kg/m^2^ are scarce.

The aim of this study was to compare the long term efficacy of SG and RYGB in Chinese T2DM patients with BMI of 28-35 kg/m^2^ using a prospective randomized trial over 36 months post-operatively.

## Methods

We designed a prospective randomized study to determine whether SG is as effective as RYGB for T2DM remission in Chinese patients with BMI of 28-35 kg/m^2^ and a short history of disease. The study was conducted in Department of Gastrointestinal Surgery of the 1^st^ affiliated hospital and Jihua hospital of Jinan University, Guangzhou, China. The trial was conducted from July 1, 2009 through July 30, 2014. The human ethics committee of Jinan University approved and supervised the whole study.

### Patients

**Fig. 1:**
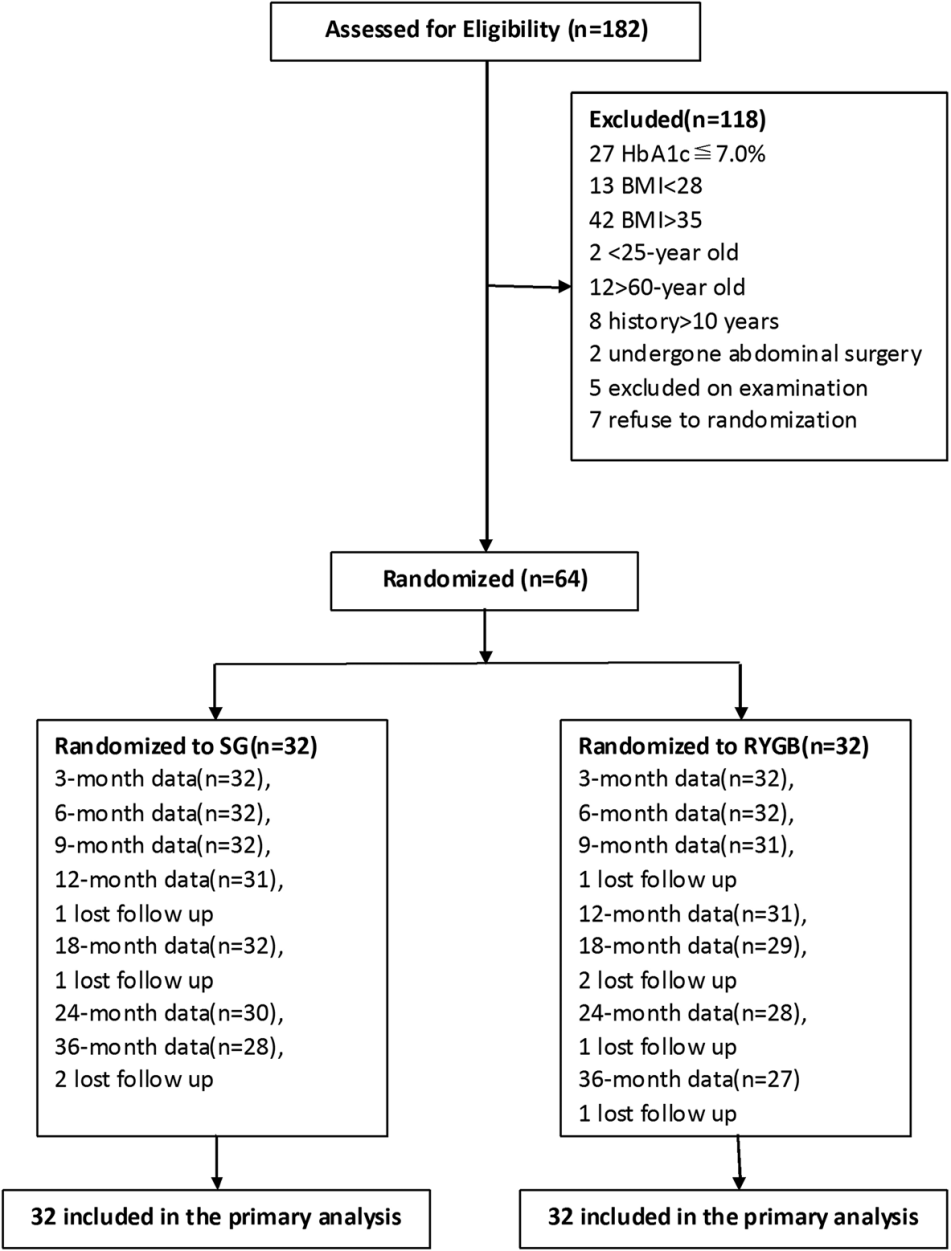
Flow diagram of patients in the study

Sixty-four patients enrolled in this study. Inclusion criteria included: (a) diagnosis of poorly controlled T2DM after 6 months medicine therapy [glycated hemoglobin A1c (HbA1c) level ≥7.0 %], (b) measured BMI of ≥28 and ≤ 35 kg/m2, (c) aged 25 to 60 years old, (d) diabetes duration of less than ten years, and (e) patients were excluded if they had undergone previous bariatric surgery or other complex abdominal surgery or if they had poorly controlled medical problems. Patients were also excluded if they had C-peptide levels below 0.8 ng/ml. In addition to the assessments for inclusion, each patient was assessed for their general condition and mental status, complications of obesity and diabetes mellitus, risk factors, and motivations for surgery (Fig.1). A computer-generated variable block schedule was used for randomization. Allocation to treatments was not concealed and patients knew which procedure they were to undergo.

### Surgical interventions

In order to reduce the differences in surgical techniques, the same team and the same physician (Cunchuan Wang) carried out the operations for both groups. Described briefly, the surgical methods were as follows: For laparoscopic SG, four trocars were placed and 38 Fr. Bougie was used to calibrate the sleeve. The greater curvature was cut out 4 cm from the pylorus using a linear stapler towards His angle to completely remove the fundus of the stomach. The remnant gastric cutting edge was 2 cm from the lesser curvature of stomach. Then, the cutting edge was continuously sutured with 3-0 absorbable sutures, which are good for hemostasis. For laparoscopic RYGB, 5 trocars were used. The volume of gastric pouch was approximate 10-20 ml. The length of the biliopancreatic limb was 25 cm, and the Roux limb was 125 cm. The anastomotic stoma between stomach and jejunum was 1.5 cm and 6 cm between jejuna.

During the operation, no routine stomach and drainage tubes were placed. Patients fasted the first day post-operation and followed a complete liquid and a soft diet for one month. Subsequently, patients followed a half liquid diet for three months and gradually arrived at a general diet. The patients received follow-up examinations in an outpatient clinic, Patients took a proton pump inhibitor and gastric mucosa protective agent for six weeks post-operation. In addition, the patients routinely took multivitamin supplementation and calcium tablet for a long period. The vitamin status was not checked regularly.

### Follow up and data collection

In one year post operation, the patients attended the visit every three months, and half-yearly thereafter. We collected the patients’ height, body weight, BMI, waist circumference, usage of medication and adverse events. The laboratory test included HbA1c, FBG, C-peptide, and serum lipid profiles.

### Study end points

The primary outcome was glycemic control with HbA1c values less than 6.0 % in addition to fasting plasma glucose levels less than 7.0 mmol/L without glycemic agents at the 36-month visit. Secondary outcome measures included the percentage of weight loss and improvement of dyslipidemia. Any adverse events were also recorded.

### Statistical analysis

As previous study has shown a remission rate in RYGB group of 80 % [12], we assumed that SG would lead to a lower remission rate of 40 % in the lower BMI patients. Using a sample size of 64 patients (32 per group), we would have had the power to detect this difference with an ɑ level of 0.05 and power of 90 %.

All analyses were performed using SPSS 17.0 (SPSS Inc., Chicago, Illinois). Chi-square and t-tests were used to compare differences between two groups. Continuous variables were reported as means with standard deviation. A 2-sided P value of <0.05 was considered statistically significant.

## Results

### Patient characteristics

Nine (14.1 %) patients failed to finish the whole 36 months follow-up, and this included four from SG group and five from RYGB group. The patients’ characteristics at baseline are summarized in Table 1. Both groups had similar baseline anthropometric measurements, including age, gender, weight, height, BMI, waist circumference, duration of diabetes, and medication usage conditions (Table 1). Baseline values of HbA1c (8.5 % vs. 8.9 %, *P* = 0.321), FBG (10.2 vs. 10.4 mmol/L, *P* = 0.700), and C-peptide (2.2 vs. 2.6 ng/ml, *P* = 0.062) in the SG group were comparable to the RYGB group. The two groups also had similar baseline serum lipid levels that included cholesterol, triglyceride, HDL, and LDL.

**Table 1:** Baseline patients characteristics

Characteristic	SG (*n* = 32)	RYGB (*n* = 32)	*P* value
Demographic, mean (SD)			
Age (yrs)	40.4 ± 9.4	41.4 ± 9.3	0.681
Sex, female-no.(%)	23 (71.9)	19 (59.4)	0.292
Height (cm)	166.8 ± 6.8	170.3 ± 8.6	0.077
Weight (kg)	88.4 ± 6.8	94.3 ± 13.3	0.055
Body mass index (kg/m^2^)	31.8 ± 3.0	32.3 ± 2.4	0.374
Waist circumference (cm)	103.0 ± 7.7	104.5 ± 6.8	0.404
Duration of diabetes (yrs)	4.0 ± 1.7	4.2 ± 1.9	0.710
Glycemia, mean (SD)			
HbA1c (%)	8.5 ± 1.2	8.9 ± 1.3	0.321
FBG (mmol/L)	10.2 ± 2.7	10.4 ± 2.2	0.700
C-peptide (ng/ml)	2.2 ± 0.7	2.6 ± 1.0	0.062
Serum lipids, mean (SD)			
Cholesterol (mmol/L)	5.0 ± 1.1	4.6 ± 0.9	0.092
Triglyceride (mmol/L)	3.2 ± 1.7	3.0 ± 2.0	0.545
HDL (mmol/L)	1.1 ± 0.2	1.0 ± 0.1	0.067
LDL (mmol/L)	3.8 ± 1.1	3.9 ± 0.9	0.702
Medication usage-no.(%)			
Oral hypoglycemic	31 (96.9)	30 (93.8)	0.554
Insulin usage	15 (46.9)	18 (56.2)	0.453
Antihypertension	10 (31.2)	12 (37.5)	0.599
Lipid-lowering drug	21 (65.6)	18 (56.2)	0.442

### Surgical treatments and complications

All procedures were successfully performed by laparoscopic techniques. The surgical time was shorter for the SG group than the RYGB group (58.0 vs.103.8 mins, *P* = 0.000). The mean post-operative hospital stay was 5.2 days for the SG group and 6.6 days for the RYGB group (*P* = 0.000). There were no deaths or major complications in either group. Minor complications occurred in 3 of 55 patients (5.5 %), including 2 gastroesophageal reflux cases in the SG group and 1 case of anemia in the RYGB group. All cases with complications were resolved with medications. The case with anemia was cured with ferralia and vitamin B12 for a long term.

### Treatment effects

Primary and secondary outcomes at 36 months are shown in Table 2. 22 patients (78.6 %) in SG group and 23 patients (85.2 %) in RYGB group achieved complete remission of diabetes mellitus with HbA1c < 6.0 % (*P* = 0.525) and without taking antidiabetic medications, and 25 patients in each group (89.3 % vs. 92.6 %) gained successful treatment of diabetes with HbA1c ≤ 6.5 % (*P* = 0.100). Meanwhile, at study end point, 27 patients in SG group and 28 in RYGB group stopped receiving oral hypoglycemic agents, and 13 patients in the SG group and 18 patients in the RYGB group no longer needed insulin injections.

**Table 2:** Outcomes at 36 months

Variable	SG (28)	RYGB (27)	*P* Value
Primary outcome-no.(%)			
HbA1c ≤ 6.5 % without medications	25 (89.3)	25 (92.6)	1.000
HbA1c ≤ 6.5 % with medication	2 (7.1)	1 (3.7)	1.000
HbA1c < 6.0 % without medications	22 (78.6)	23 (85.2)	0.525
HbA1c < 6.0 % with medications	1 (3.6)	0 (0)	1.000
Glycemia, mean (SD)			
HbA1c (%)	5.9 ± 0.7	5.7 ± 0.7	0.334
Change from baseline (%)	2.7 ± 1.1	3.1 ± 1.3	0.175
FBG (mmol/L)	5.9 ± 0.7	5.8 ± 0.7	0.371
Change from baseline (mmol/L)	4.3 ± 2.7	4.8 ± 2.0	0.448
C-peptide (ng/mL)	1.7 ± 0.5	1.8 ± 0.6	0.285
Change from baseline (ng/mL)	0.5 ± 0.5	0.7 ± 0.4	0.060
Weight, mean (SD)			
%TWL	27.1 ± 7.1	31.0 ± 7.1	0.049
%EWL	81.9 ± 14.0	92.3 ± 10.5	0.003
Weight (kg)	63.3 ± 7.9	64.4 ± 8.9	0.610
Change from baseline (kg)	24.3 ± 6.5	29.5 ± 8.9	0.017
BMI (kg/m^2^)	22.8 ± 1.7	22.0 ± 1.1	0.032
Change from baseline (kg/m^2^)	9.1 ± 2.7	11.0 ± 3.2	0.017
Waist circumference (cm)	81.2 ± 3.6	79.2 ± 3.1	0.029
Change from baseline (cm)	21.6 ± 10.8	25.0 ± 6.3	0.166
Serum lipids, mean (SD)			
Cholesterol (mmol/L)	3.9 ± 0.7	3.8 ± 0.8	0.674
Triglyceride (mmol/L)	1.5 ± 0.6	1.4 ± 0.6	0.310
HDL (mmol/L)	1.5 ± 0.3	1.7 ± 0.4	0.105
LDL (mmol/L)	2.2 ± 0.7	1.9 ± 0.7	0.120
Medication usage-no.(%)			
Oral hypoglycemic agents	4 (14.3)	2 (7.4)	0.700
Insulin usage	2 (7.1)	0	0.488
Antihypertension agent	5 (17.9)	3 (11.1)	0.744
Lipid-lowering drug	3 (10.7)	1 (3.7)	0.630

**Fig. 2:**
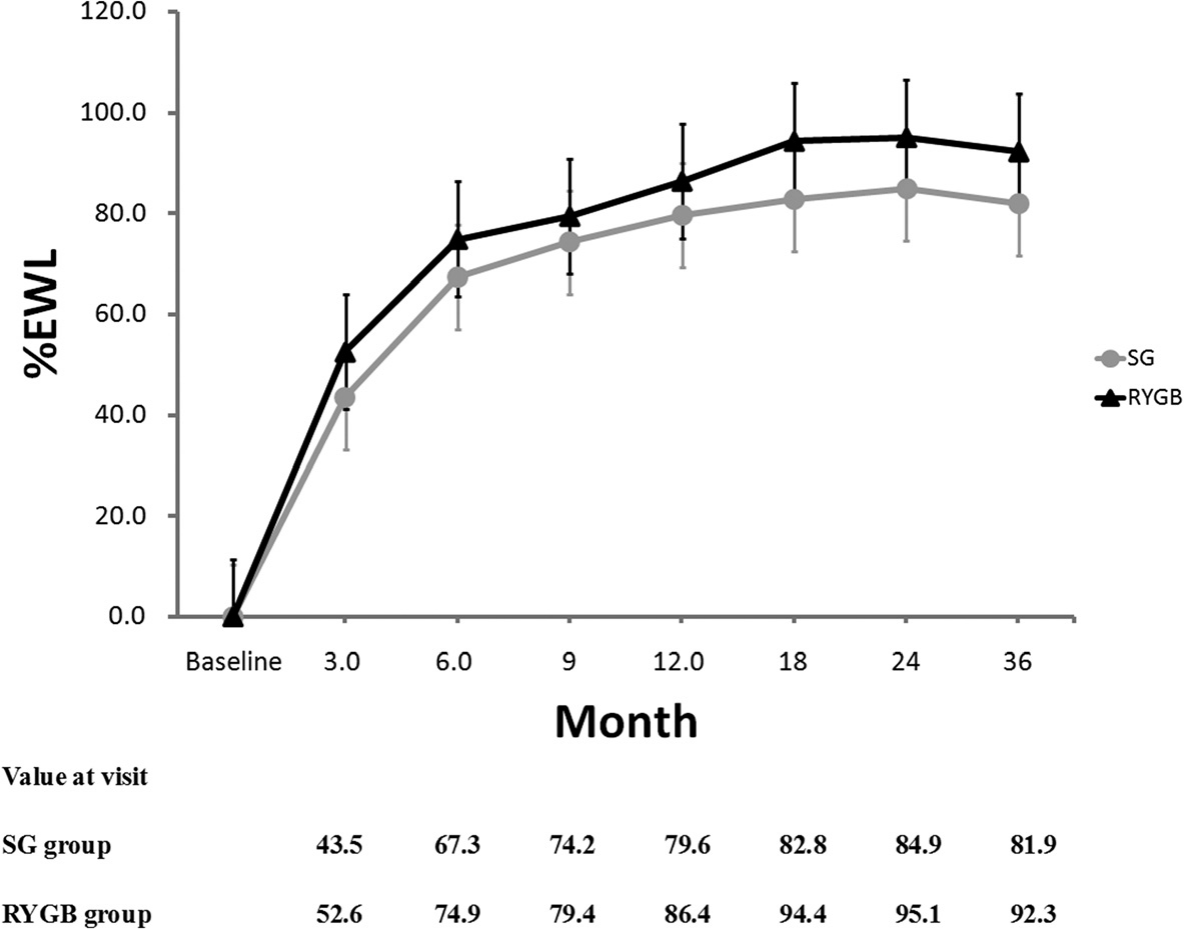
Percentage of excess weight loss are plotted for the 3, 6, 9, 12, 18, 24 and 36 month time points. Error bars indicate 95 % CIs; *P* values for differences are all <0.05

 Each group had significant weight loss compared to baseline in the follow-up. At each visit time, percentage of total weight loss (%TWL), %EWL and change in BMI were greater in the RYGB group compared to the SG group. The most weight loss time point was two-year post operation in both groups, and after that maintained the weight reduction outcomes (Fig. 2).

**Fig. 3:**
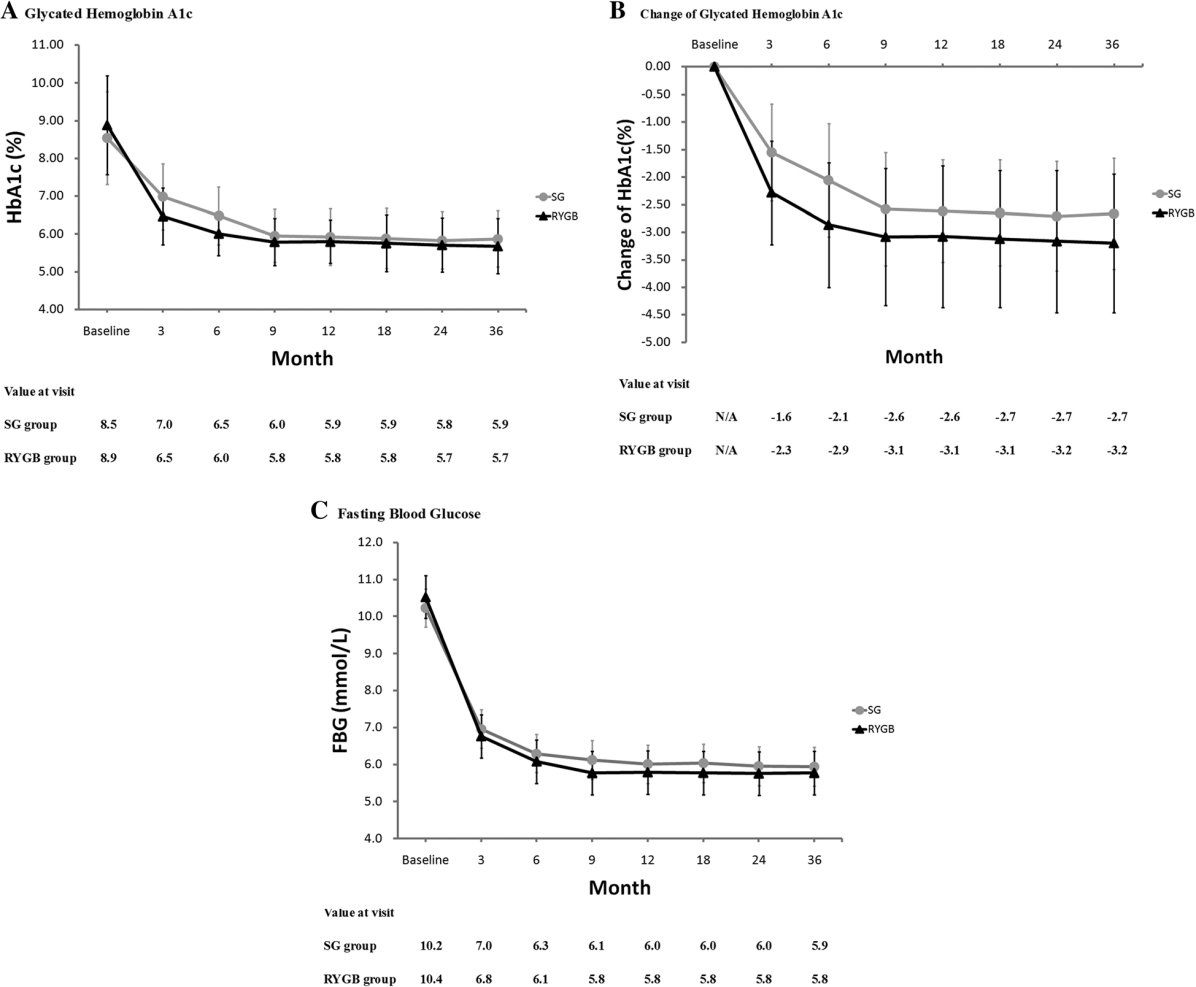
Values of HbA1c% (**a**), changes of HbA1c% (**b**) and fasting blood glucose (**c**) are plotted for the 3, 6, 9, 12, 18, 24 and 36 month time points. Error bars indicate 95 % CIs; *P* values for differences are all < 0.05

At three-year post operation, HbA1c were similar in the two study groups (5.9 vs. 5.7 mmol/L, *P* = 0.334). At 3-month and 6-month visiting post operation, HbA1c values were much lower for RYGB group than SG group, and meanwhile reductions of HbA1c were more significant for RYGB group (*P* < 0.01). After that, the values of HbA1c and changes of HbA1c were similar in the two groups (*P* > 0.05) (Fig. 3ab). FBG levels were comparable for the SG and RYGB groups at all-time points (Fig. 3c ). In both groups, HbA1c and FBG levels were significantly improved after 3 months (*P* < 0.05), and the improvements were maintained through the 36-month evaluation.

Compared to the baseline, post-operative serum lipid levels in each group were significantly improved. The serum levels of cholesterol, triglyceride, HDL, and LDL were similar at each time point for the SG group compared to the RYGB group. 35 patients (18 from SG group, and 17 from RYGB group) no longer needed lipid-lowering medications and 14 patients (5 from SG and 9 from RYGB group) no longer needed antihypertensive medications at the 36-month follow-up.

## Discussion

Bariatric surgery has favorable effects on obesity and related metabolic problems. Of available procedures, Roux-en-Y gastric bypass is a common choice. For T2DM patients with severe obesity and BMI over 35 kg/m^2^, a large number of studies have shown that both sleeve gastrectomy and RYGB procedures have favorable effects [5, 6, 13, 14]. As for T2DM patients with mild obesity, gastric bypass surgery has also shown to be effective. However, it is still controversial whether sleeve gastrectomy has the same effect for the lower BMI patients [10, 15, 16].

In Asian and Chinese populations, obesity related health risks are observed in people with BMI as low as 22 to 23 [17]. Compared to Caucasians with the same BMI, Chinese populations have significantly higher levels of subcutaneous and visceral fat, which corresponds to higher risk of cardiovascular and metabolic diseases. Thus, BMI used for diagnosing obesity in Asian and Chinese populations should be lower than in western populations [18, 19]. The Asian branch of ASMBS suggests that when treating T2DM patients with bariatric surgery, BMI should be lowered appropriately, and T2DM patients with BMI over 28 kg/m^2^ should be enrolled in clinical studies [20]. To our knowledge, there are rare studies onT2DM patients with BMI of 28-35 kg/m^2^ in the Chinese mainland. At the same time, more studies have shown that early bariatric surgical intervention can enhance the remission rates of T2DM [21, 22]. Therefore, the subjects in this study are mildly obese T2DM patients with BMI of 28-35 kg/m^2^ and disease histories of less than 10 years.

The results of this study show that three years after operation, both SG and RYGB procedures were effective in weight reduction and remission of T2DM. RYGB had significantly better effects on %TWL, %EWL, and BMI change when compared with SG, which is consistent with previous studies [23, 24]. Moreover, patients in both groups had normal BMI and achieved ideal weights one year after operation without major complications. Three years after operation, the complete T2DM remission rates (HbA1c < 6.0 % without taking anti-diabetic medicines) were 78.6 % in the SG group and 85.2 % in the RYGB group. The average HbA1c and FBG levels in both groups reached normal levels, indicating that the effects of SG were equivalent to RYGB in mildly obese T2DM patients. This is consistent with the previous prospective study from Andrei Keidar and the retrospective study from Sylvie Pham with the patients of BMI > 35 kg/m^2^ [13, 14]. However, a research outcomes from Lee et al. suggested that RYGB achieved better blood glucose control compared to SG at one and five years post-operation for the T2DM patients with BMI 25-35 kg/m^2^ [10, 16]. In Lee’s study, BMI of the patients was relatively lower and the diabetic history was longer (RYGB 5.8 years vs. SG 6.9 years). These factors may have caused the patients to be more pancreatic insufficient than peripheral insulin resistance, and that may cause the lower remission rate of T2DM.

Additionally, our study showed that in both groups, all blood lipid indexes were significantly decreased after operation in the patients with dyslipidemia. Three years after operation, the blood lipid indexes, including total cholesterol, triglyceride, LDL, and HDL, stayed at normal levels with similar degrees of decline. Meanwhile, the percentages of patients that stopped taking lipid-lowering drugs and antihypertensive drugs were the same, illustrating that both SG and RYGB have similar effects on obesity relevant metabolic disturbances.

Even now, the mechanism through which bariatric surgery treats T2DM is unclear. This study investigates clinical effects but not the underling mechanism. We can see from this study, RYGB gained more significant HbA1c reduction than SG in the first 6 months after operation, and that implied RYGB improves more rapidly for T2DM. Because the RYGB operation bypasses the proximal intestine, hypotheses of its mechanism include the Ghrelin hypothesis, hindgut hypothesis, and foregut hypothesis [25, 26]. After the SG operation, insulin resistance was obviously alleviated, while the incretin hormones level was significantly increased [26–29]. Peterli et al. found that one year after surgery, RYGB ghrelin levels approached preoperative values while SG ghrelin levels were still markedly attenuated. Meanwhile cholecystokinin concentrations after test meals increased less in the RYGB group than in the SG group. They suggested that bypassing the foregut is not the only mechanism responsible for improved glucose homeostasis [30]. Schauer PR et al. concluded that weight loss and a shorter duration of diabetes were the main predictors of having a glycated hemoglobin level of 6.0 % or less after surgery [31]. Our study suggests that both groups obtained similar diabetic remission rate but different weight loss effects 3 years after operation. The relationship between glycemic control and weight loss needs to be further investigated.

This comparative study on clinical effects has some limitations that include lack of data collection on insulin resistance alleviation degree and lack of gastrointestinal GLP-1, GIP, and PYY hormones data collection. These data would help to determine the surgical mechanism for T2DM resolution in Chinese patients with BMI of 28-35 kg/m^2^. In addition, three-year follow-up is not long enough to assure that RYGB or SG can completely alleviate T2DM. Therefore, a longer period of follow-up is required.

## Conclusion

Through three-year clinical data analysis, it can be concluded that for Chinese mildly obese T2DM patients with BMI of 28-35 kg/m^2^, SG had similar effects to RYGB in remission of T2DM and metabolic disorders, but a longer follow-up period is still required to confirm the long-term effects.

## Abbreviations

SG: Sleeve gastrectomy
RYGB: Roux-en-Y gastric bypass
T2DM: Type 2 diabetes mellitus
BMI: Body mass index
HbA1c: Hemoglobin A1c
%EWL: Percentage of excess weight loss
FBG: Fasting blood glucose
%TWL: Percentage of total weight loss
HDL: high density lipoprotein
LDL: low density lipoprotein
GLP-1: Glucagon-like peptide 1
GIP: Gastric inhibitory polypeptide
PYY: Peptide YY
